# Self‐Management Intentions and Behaviors Among CKD Patients at Predialysis and Dialysis Stages: A Cross‐Sectional Study Based on Protection Motivation Theory

**DOI:** 10.1155/nrp/6668409

**Published:** 2026-05-19

**Authors:** Dayu Tang, Min Liang, Wenyi Wang, Yan Liang

**Affiliations:** ^1^ School of Nursing, Fudan University, Shanghai, 200032, China, fudan.edu.cn; ^2^ School of Social Development and Public Policy, Fudan University, Shanghai, 200032, China, fudan.edu.cn

**Keywords:** behavior, chronic kidney disease, intention, protection motivation theory, renal nursing, self-management

## Abstract

**Aim:**

To explore factors and pathways of self‐management intentions and behaviors in patients with chronic kidney disease at predialysis and dialysis stages using protection motivation theory.

**Design:**

A cross‐sectional study.

**Methods:**

This cross‐sectional study was conducted in Changning District, Shanghai, China. A total of 550 patients with CKD (216 dialysis and 334 predialysis) were recruited from local healthcare institutions via self‐administered questionnaires. Self‐management behaviors were assessed using stage‐appropriate instruments validated for predialysis and dialysis populations, respectively. Protection motivation theory constructs were measured and analyzed using confirmatory factor analysis and structural equation modeling to explore pathways across groups.

**Results:**

The structural equation model demonstrated acceptable fit (*χ* ^2^/df = 3.052, CFI = 0.903, RMSEA = 0.054). Compared with predialysis patients, dialysis patients reported lower perceived severity but higher response efficacy and self‐management intention. Self‐efficacy and response efficacy were positively associated with intention. Furthermore, perceived vulnerability, self‐efficacy, and intention were positively related to self‐management behavior.

**Conclusion:**

Response efficacy emerged as a key factor associated with self‐management intention. These findings suggest that nursing interventions should prioritize demonstrating the effectiveness of self‐management behaviors. However, as this was a cross‐sectional study conducted in a single district, causal inferences should be drawn with caution.

**Implications for the profession and patient care:**

Healthcare providers should transition to stage‐tailored cognitive interventions. For predialysis patients, education must tangibilize the benefits of early behavioral activation. For dialysis patients, nurses should implement concrete “efficacy‐focused feedback loops”—such as explicitly linking a patient’s routine objective data (e.g., stabilized lab results or reduced interdialytic weight gain) directly to their adherence efforts—to reinforce response efficacy. Furthermore, policymakers should champion standardized, efficacy‐driven CKD management frameworks adaptable across hospital and community settings.

**Patient or Public Contribution:**

Patients and healthcare staff contributed to the study design and recruitment process. The Shanghai Municipal Health Commission of Changning District assisted with participant recruitment, while staff from the Nephrology Departments of Tongren Hospital and Zhongshan Hospital provided essential support. Their involvement shaped the study’s data collection and ensured that the findings were relevant to clinical practice.

**Impact**
• This study advances behavioral science in nephrology by revealing a structural motivational shift: The “engine” of self‐management evolves from being threat‐driven in the predialysis stage to efficacy‐driven in the dialysis stage.
• By addressing critical evidence gaps, the findings provide a data‐driven rationale for moving beyond generic health education, equipping clinicians with actionable, stage‐specific strategies to sustain long‐term treatment adherence.

## 1. Introduction

Chronic kidney disease (CKD) affects over 750 million individuals worldwide [1, 2], accounting for 8.7 million years lived with disability and ranking among the top contributors to global nonfatal disease burden [3, 4]. This immense clinical burden translates into complex, daily self‐care challenges for patients, often leading to treatment fatigue and nonadherence. As an irreversible, progressive condition, CKD requires rigorous, sustained self‐management. However, the nature of this self‐management evolves significantly as the disease progresses: Predialysis patients primarily focus on dietary modifications, blood pressure control, and medication adherence to delay disease progression, whereas dialysis patients must adhere to highly demanding, survival‐dependent regimens, including strict fluid restriction and vascular access care [5–9]. As patients transition from the often‐asymptomatic predialysis stage to the highly demanding dialysis stage, their cognitive appraisals of the disease inevitably shift. Yet, how stage‐specific motivational mechanisms shape differing self‐management intentions and behaviors across these stages remains unclear.

Recent systematic reviews highlight that existing research remains largely fragmented [10, 11]. Studies have predominantly focused on isolated self‐management behaviors (e.g., medication adherence, dietary control, and physical activity), or on psychosocial correlates of these behaviors, often within a single disease stage, particularly among patients receiving dialysis [8, 12, 13]. Consequently, relatively little attention has been paid to how underlying motivational mechanisms dynamically shift across the CKD trajectory. This fragmented approach limits the development of holistic, evidence‐based, and stage‐tailored nursing interventions.

Applying a robust theoretical framework can provide valuable insights into these shifting mechanisms. Protection motivation theory (PMT), a widely used social cognitive model [14], offers a highly relevant structure for progressive chronic illnesses. While other models, such as the theory of planned behavior, focus primarily on attitudes and social norms, PMT uniquely enables the simultaneous examination of fear‐driven threat appraisals (perceived severity and vulnerability) and efficacy‐driven coping appraisals (response efficacy and self‐efficacy). This dual‐pathway approach is particularly suited for CKD, where patients must constantly balance the fear of disease progression with their practical capacity to execute complex care tasks. Although PMT has proven effective in predicting health behaviors in diverse contexts [15–18], its application to CKD self‐management—particularly in comparing stage‐specific behaviors—remains underdeveloped [19].

Therefore, this study aims to bridge these literature gaps by leveraging PMT to examine the motivational factors influencing self‐management intention and behavior across predialysis and dialysis stages. By elucidating these pathways, this study seeks to provide actionable insights for stage‐tailored renal nursing strategies, such as targeted patient education, counseling approaches, and behavioral coaching to enhance sustained self‐management adherence across the CKD trajectory.

## 2. Background

Self‐management in CKD is highly complex, requiring patients to actively navigate severe dietary restrictions (e.g., controlling protein, potassium, and phosphorus), adhere to complex polypharmacy, rigorously monitor fluid intake, and manage progressive symptoms [5]. While theories such as social cognitive theory have been widely used to explain self‐management, they often emphasize self‐efficacy while lacking a robust mechanism to evaluate how the fear of disease progression interacts with coping mechanisms over a deteriorating disease trajectory [17, 20]. In contrast, PMT [21] integrates threat appraisal (perceived severity and vulnerability) and coping appraisal (response efficacy and self‐efficacy). Empirical evidence suggests that PMT is uniquely suited for progressive chronic illnesses because it captures the tension between fear‐driven motives and action‐driven capabilities. In real‐world CKD management, PMT constructs are highly operational: perceived severity reflects the fear of requiring dialysis or experiencing cardiovascular events, while response efficacy captures the patient’s belief that adhering to a restricted diet will actually delay renal failure [22–24].

Although traditional self‐management interventions improve general outcomes, they frequently fail to enhance patient activation, particularly in advanced CKD [8, 25]. This failure often occurs because standard interventions rely heavily on generic knowledge transfer, neglecting the profound motivational burnout and fatalism that develop as the disease worsens [26]. Consequently, clinical pathways frequently treat CKD patients as a homogeneous group, applying a “one‐size‐fits‐all” approach that ignores stage‐specific psychological shifts. Mapped onto PMT, predialysis patients—who are often asymptomatic—may exhibit low threat appraisal (perceived severity), leading to poor dietary compliance. Conversely, dialysis patients face immediate, life‐threatening severity but may experience diminished coping appraisal (self‐efficacy) due to the overwhelming burden of the dialysis regimen. Because comparative studies utilizing unified theoretical frameworks across CKD stages are exceedingly rare, a critical evidence gap remains, limiting the development of stage‐tailored guidelines.

Accordingly, this study applies PMT to examine whether and how the relative roles of threat appraisal and coping appraisal differ between predialysis and dialysis patients. Understanding these stage‐specific mechanisms will guide the design of differentiated nursing interventions, such as targeted threat education for early stages and efficacy‐enhancement support for advanced stages.

## 3. Study

### 3.1. Aim

The aim of this study is to investigate the factors influencing self‐management intention and behavior among patients with CKD at the predialysis and dialysis stages using PMT, thereby uncovering structural pathways to inform stage‐specific interventions.

### 3.2. Objective

A. To identify the levels of cognitive and motivational factors (threat and coping appraisals) influencing self‐management intention and behavior in CKD patients.
B. To evaluate differences in PMT constructs and self‐management intentions between predialysis and dialysis patients, adjusting for relevant sociodemographic and clinical covariates.
C. To analyze the structural pathways through which PMT constructs predict self‐management intention and subsequent behavior.
D. To provide data‐driven recommendations for healthcare providers to design stage‐specific interventions.

### 3.3. Research Question

RQ1: How do the levels of PMT constructs (threat appraisal, coping appraisal) and self‐management intention differ between patients in the predialysis and dialysis stages?

RQ2: What are the structural pathways through which PMT constructs influence self‐management intention and behavior across the CKD trajectory?

## 4. Methods

### 4.1. Research Design

This study adopted a cross‐sectional design. While a longitudinal approach would ideally capture the evolution of self‐management, a cross‐sectional design was selected as a necessary foundational step to validate the theoretically hypothesized structural relationships of PMT across CKD stages before investing in longitudinal cohorts. The analysis was drawn from a larger research project examining CKD management from the perspectives of both primary and specialist health professionals and patients. In this article, patients’ data were encrypted, processed, and utilized in testing the structural relationships between stages of illness, the PMT constructs, and self‐management behaviors.

### 4.2. Study Setting and Sampling

This study was conducted in Changning District in Shanghai, as representative of a generalist–specialist transfer model of the CKD management system, and included all public healthcare institutions providing CKD management service in Changning District (i.e., a tertiary hospital, a secondary hospital, and 10 community health service centers). Participants were recruited using convenience sampling due to resource and logistic constraints, with dialysis patients recruited from hospital settings and predialysis patients from community health centers. While this sampling strategy mirrors the actual tiered healthcare delivery system in Shanghai—thereby enhancing ecological validity—we acknowledge that it may introduce selection bias regarding healthcare exposure. Furthermore, as the setting is limited to an urban district, findings may not fully generalize to rural or resource‐limited settings.

A structural equation modeling (SEM)–based power analysis was conducted to determine the minimum sample size. Based on the given latent variables, the number of observed variables, a power of 0.90, an effect size of 0.3, and an alpha level of 0.05, a minimum sample size of 175 for each group (dialysis and nondialysis) was required. The final sample of 550 comfortably exceeded this requirement and satisfied the SEM heuristic of 10–20 cases per estimated parameter.

### 4.3. Inclusion and Exclusion Criteria

Participants included patients who met the following inclusion criteria: (1) age ≥ 18 years, (2) diagnosed with CKD for more than 3 months (based on medical records), with and without dialysis, and (3) intact cognition (verified via the absence of cognitive impairment in medical records during a prescreening by the attending physician), able to communicate, and willing to provide informed consent and participate in the study. Exclusion criteria were (1) physical disabilities (e.g., severe visual or hearing impairment) or acute clinical instability that precluded independent completion of a paper‐based survey and (2) diagnosed dementia or intellectual disabilities. While excluding sensory‐impaired individuals was a pragmatic necessity, we acknowledge that this limits the representativeness of this vulnerable subgroup.

### 4.4. Data Source

Self‐management behaviors were assessed using the revised chronic kidney disease self‐management (CKD‐SM) questionnaire for patients in the predialysis stage [27, 28] and the self‐management behavior scale for hemodialysis patients (SBSHP) for patients in the dialysis stage [29]. The CKD‐SM assesses four domains using 29 items (problem‐solving, self‐integration, seeking social support, and adherence to recommended regimens), while the SBSHP includes 25 items evaluating behaviors specific to dialysis, such as fluid and ion restriction, dietary management, and psychosocial adjustment. Items in both scales are rated from 1 (*never*) to 4 (*always*). To pragmatically address the discrepancy in scale length (29 vs. 25 items), the item mean score was calculated to normalize both outcomes onto a comparable 1‐to‐4 metric. However, we acknowledge that this mathematical adjustment does not resolve the inherent issue of measurement nonequivalence. Because the two scales evaluate qualitatively different self‐management domains tailored to specific clinical realities, strict psychometric comparability of absolute self‐management levels across the predialysis and dialysis groups is inherently constrained. Consequently, in our SEM analyses, our objective was not to directly compare raw outcome scores, but rather to evaluate and compare the structural predictive pathways—specifically, how PMT cognitive constructs drive self‐management behaviors within their respective clinical contexts.

Item generation (see Supporting Table S1) was strictly guided by Rogers’ PMT theoretical framework, adapted using existing chronic illness literature [29–32]. Content validity was assessed by a panel of five experts (senior nephrology nurses and health psychologists, all with > 10 years of experience). The Item‐Level Content Validity Index (I‐CVI) was calculated, and items were revised to achieve an I‐CVI ≥ 0.80 for relevance and clarity.

Confirmatory factor analysis (CFA) confirmed an acceptable five‐factor model fit. During this process, no cross‐loadings were permitted. However, we applied specific model modifications to refine the factor structure: three items (vuln1, vuln2, and self1) were removed due to poor factor performance, and four pairs of error terms were allowed to correlate based on modification indices and semantic similarities (inte2 with inte1; resf4 with resf3; seve4 with seve2; and inte1 with self2). To ensure the best possible measurement model, alternative structures were systematically compared (see Supporting Table S3). We evaluated both the initial full model (Model A) and the refined item‐reduced model (Model B), both with and without the correlated error terms. By comparing fit indices and information criteria across these four alternatives, the final modified model (Model B—after error correlation) yielded the most satisfactory fit (*χ* ^2^(138) = 372.738, CFI = 0.920, TLI = 0.901, RMSEA = 0.056) and was adopted for subsequent SEM. The standardized factor loadings of the observed variables in this final model ranged from 0.479 to 0.877 (Table 1); although one item (Inte2) loaded slightly below 0.50, it was retained due to its critical theoretical importance regarding exercise intention in PMT. The Cronbach’s alpha ranged from 0.712 to 0.861, indicating an acceptable result.

**TABLE 1:** Confirmatory factor analysis and factor loadings.

Five factors and scale items	Standardized loading
F1: Perceived severity/Seve: Cronbach’s *α* = 0.747	
Decreased kidney function is a serious problem for me. (Seve1)	0.576
If I don’t maintain treatment, my condition may relapse or worsen again even after it gets better. (Seve2)	0.583
Renal failure and complications will increase my family’s burden and lead to poverty. (Seve3)	0.684
If my condition continues to deteriorate, it will be difficult for me to do anything. (Seve4)	0.742
F2: Perceived vulnerability/Vuln: Cronbach’s *α* = 0.763	
The likelihood of my relapse is increased with insufficient exercise. (Vuln3)	0.703
The likelihood of my disease deterioration is increased with bad mood and anxiety. (Vuln4)	0.877
F3: Self‐efficacy/SelfEff: Cronbach’s *α* = 0.861	
I can take medication as prescribed and self‐monitor and perform self‐care. (Self2)	0.640
I can remain optimistic about treatment of the disease. (Self3)	0.795
When the disease recurs or worsens, I can find ways to cope with it with the support of my doctor. (Self4)	0.822
I can prevent complications effectively. (Self5)	0.716
I can take the initiative to communicate and interact with doctors to help me manage my kidney disease. (Self6)	0.774
F4: Response efficacy/RespEff: Cronbach’s *α* = 0.784	
Knowledge of the disease can make me more adherent to treatment. (Resf1)	0.662
Following a low‐protein diet as directed by my doctor can improve my quality of life. (Resf2)	0.642
Getting support from my family and friends can benefit treatment of the disease. (Resf3)	0.652
With regular follow‐up and medication, my condition can be controlled to an ideal state. (Resf4)	0.732
F5: Intention (Perceived protection motivation)/Inte: Cronbach’s *α* = 0.712	
I plan to take my medications as recommended by my doctor. (Inte1)	0.516
I plan to do a moderate amount of exercise (the amount recommended by my doctor) or rehabilitation activities each week. (Inte2)	0.479
I plan to gain more knowledge about the disease (through doctors, books, and the internet). (Inte3)	0.734
I plan to strictly control my diet and water intake in order to slow down the progression of my disease and improve my quality of life. (Inte4)	0.678

*Note:* All standardized factor loadings are significant at *p* < 0.001.

Five factors were identified and named perceived severity, perceived vulnerability, self‐efficacy, response efficacy, and intention. Factor scores for perceived severity, perceived vulnerability, self‐efficacy, response efficacy, and intention were used to represent the degree of each PMT subconstruct, respectively. The higher the scores, the higher degree of each PMT subconstruct.

As the predialysis and dialysis stages were focused in this study, whether a patient was currently receiving dialysis was used to represent the predialysis and dialysis phases. Participants who were diagnosed with CKD for over 3 months (based on medical records) and reported not having undergone dialysis were categorized as in the predialysis stage (coded as “0,” *n* = 334). Patients who were receiving dialysis were categorized as being in the dialysis stage (coded as “1,” *n* = 216). The researcher explored alternative approaches to code “dialysis stage” (=1) as “Stage 5 (complete renal failure) and receiving dialysis currently” (*n* = 216) and “predialysis stage” (=0) as “Stage 1–4 and not receiving dialysis currently” (*n* = 310), excluding 24 who were in Stage 5 (complete renal failure) but not receiving dialysis currently, and performed a sensitivity analysis using this alternative coding scheme. This approach was utilized to isolate Stage 5 nondialysis patients, as their psychological profile (being on the immediate brink of dialysis) might uniquely confound the broader predialysis group.

Participants’ sociodemographic characteristics were used as control variables and assessed in five aspects: (1) age (in years); (2) sex (male and female); (3) marital status (married and other); (4) educational attainment (primary school or less, middle school, high school, college, and more); and (5) income (CNY per year, ≤ 5,000, 5001–10,000, 10,001–15,000, and 15,001+). These specific variables were selected based on established literature highlighting their foundational impact on health literacy and chronic disease self‐management.

### 4.5. Data Collection

An independent research assistant, strictly separate from the clinical care team, performed data collection after receiving standardized training on neutral survey administration between November 2017 and January 2018. We acknowledge the time elapsed; however, while medical treatment protocols may evolve, the fundamental human cognitive processes (threat and coping appraisals) outlined by PMT remain highly stable and consistent. Thus, the identified behavioral mechanisms retain strong relevance to current CKD management practices. To minimize social desirability bias, participants completed the anonymous surveys independently and were assured that their responses would not affect their medical care. Participation was entirely voluntary. Upon completion of the survey, participants received a nominal token of appreciation (valued at <$2 USD); this procedure was approved by the Ethics Committee as it was deemed insufficient to induce undue coercion or influence study enrollment. Of 600 eligible patients approached, 586 were surveyed, and 557 completed the questionnaire. After excluding seven incomplete surveys (listwise deletion), 550 patients were included. No systematic demographic differences were observed between completers and noncompleters.

### 4.6. Data Analysis

**FIGURE 1:**
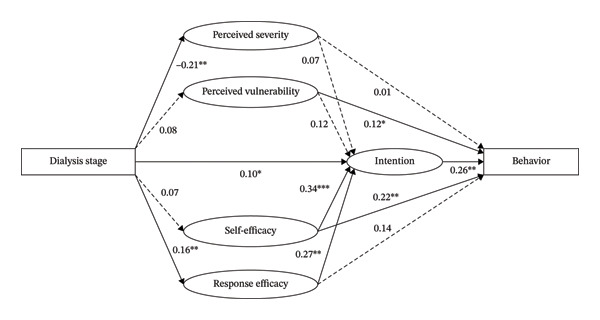
Structural model results. ^∗^ *p* < 0.05, ^∗∗^ *p* < 0.01, and ^∗∗∗^ *p* < 0.001. Dashed lines represent nonsignificant pathways.

Descriptive statistics were used to summarize sample characteristics. SEM was used to examine the relationship between stages of illness, the PMT constructs, and CKD self‐management behaviors. The hypothetical SEM (Figure 1) was tested to examine the relationships among the constructs. A maximum likelihood (ML) factor analysis with promax rotation was employed to examine the dimensionality of the outcome expectations scales. The rationale for this analytical approach is detailed in Supporting Table S2 and Figure S1. Prior to SEM, assumptions were checked; variance inflation factors (VIF < 3) confirmed the absence of multicollinearity. To address potential common method bias, Harman’s single‐factor test was conducted, revealing that a single factor accounted for less than 50% of the variance. Because some data deviated from multivariate normality, the ML with robust (MLR) standard errors estimator was utilized to obtain robust parameter estimates. Model fit was assessed using the following indexes [33]: the comparative fit index (CFI) > 0.9, Tucker–Lewis index (TLI) > 0.9, root mean square error of approximation (RMSEA) < 0.08 (generally < 0.06 in the most standard recommendations, and < 0.08 is an acceptable fit), and chi‐square/degrees of freedom (df) < 5.0.

Based on the CFA model, the hypothetical SEM (Figure 1) was performed to examine the relationships among the constructs and observed variables, by adding a binary independent variable “dialysis stage,” a continuous dependent variable “CKD self‐management behaviors,” and control variables. All analyses were performed with Mplus 8.0 (Muthen & Muthen, Los Angeles, CA, USA) and Stata SE 17.0 (StataCorp LLC, College Station, TX, USA).In summary, employing a cross‐sectional design, this study recruited 550 patients from tiered healthcare settings. Self‐management behaviors were assessed using stage‐specific validated instruments, while PMT constructs were measured via a scale rigorously validated through comparative CFA models. SEM was then utilized to examine the hypothesized structural pathways, controlling for sociodemographic covariates.

### 4.7. Ethical Consideration

This study was approved by the ethical committee of the Zhongshan Hospital, associated with Fudan University (IRB NO. B2017‐143, 11th October, 2017). Participants were asked to complete a questionnaire while awaiting their check‐up in the included healthcare institutions. Informed consent was obtained before data collection. To ensure strict confidentiality, while clinical staff assisted in verifying patient eligibility via medical records prior to recruitment, the actual survey administration was entirely anonymous. No identifying information (e.g., names or medical IDs) was recorded on the questionnaires, and all data were stored securely on encrypted devices.

## 5. Results

### 5.1. Descriptive Statistics

The characteristics of respondents are reported in Table 2. The mean participant age was 62.1 (15.5) years, 53.1% were men, 83.4% were married, 24.9% were college graduates, 37.8% reported that their income was no more than 50,000 CNY per year, and 39.3% were receiving dialysis. The dialysis group was younger than nondialysis group. No significant differences were observed between the two groups in education, marital status, and income.

**TABLE 2:** Profile of survey respondents and comparison between dialysis and nondialysis groups.

Variable	Whole sample (*n* = 550)	Dialysis stages		*p* Value
		Dialysis (*n* = 216)	Nondialysis (*n* = 334)	
Age (years)	62.1 ± 15.5	57.3 ± 0.97	65.2 ± 0.85	< 0.001
Sex				0.561
Male	292 (53.1)	118 (54.6)	174 (52.1)	
Female	258 (46.9)	98 (45.4)	160 (47.9)	
Education				0.416
(i) Primary school or less	87 (15.8)	28 (13.0)	59 (17.7)	
(ii) Middle school	161 (29.3)	64 (29.6)	97 (29.0)	
(iii) High school	165 (30.0)	71 (32.9)	94 (28.1)	
(iv) College and more	137 (24.9)	53 (24.5)	84 (25.2)	
Marital status				0.683
(i) Married	459 (83.4)	182 (84.3)	277 (82.9)	
(ii) Others	91 (16.6)	34 (15.7)	57 (17.1)	
Income (CNY/year)				
(i) ≤ 5000	208 (37.8)	85 (39.4)	123 (36.8)	
(ii) 5001–10,000	215 (39.1)	74 (34.3)	141 (42.2)	0.259
(iii) 10,001–15,000	76 (13.8)	34 (15.7)	42 (12.6)	
(iv) 15,001+	51 (9.3)	23 (10.6)	28 (8.4)	

*Note: t*‐test was used for continuous variables, and chi‐square test was used for categorical variables to explore differences between dialysis and nondialysis groups.

### 5.2. Measurement Model

CFA was performed to confirm an acceptable fit of the PMT constructs. Two alternative models (Model A and Model B, see Supporting Table S3) were examined, respectively, and the final CFA (Model B—after error correlation) showed satisfactory fit indices for the five latent variables: *χ*2(138) = 2.701, *p* < 0.001, CFI = 0.920, TLI = 0.901, and RMSEA = 0.056. The standardized factor loadings of the observed variables ranged from 0.479 to 0.877 (see Table 1). Correlations among the five latent constructs are shown in Table 3.

**TABLE 3:** Results of the measurement model.

Construct	Seve	Vuln	SelfEff	RespEff	Inte
Seve		0.432^∗∗∗^	0.139^∗^	0.379^∗∗∗^	0.256^∗∗∗^
Vuln	0.054		0.414^∗∗∗^	0.568^∗∗∗^	0.448^∗∗∗^
SelfEff	0.066	0.049		0.678^∗∗∗^	0.616^∗∗∗^
RespEff	0.076	0.048	0.045		0.642^∗∗∗^
Inte	0.066	0.052	0.048	0.049	

*Note:* Seve = perceived severity; Vuln = perceived vulnerability; SelfEff = self‐efficacy; RespEff = response efficacy; Inte = intention. Correlations among latent constructs are above the diagonal; standard errors among latent constructs are below the diagonal.

^∗∗∗^ *p* < 0.001.

### 5.3. Structural Model

After identifying a well‐fitted measurement model, the hypothesized relationships between variables in the structural model was followed to be tested with guidance of proposed conceptual framework. The results of the structural model revealed a satisfactory fit to the data (*χ* ^2^ = 680.677, df = 223, *p* < 0.001, *χ* ^2^/df = 3.052, RMSEA = 0.054 [90% CI 0.049, 0.059], CFI = 0.903, and TLI = 0.873) (see Figure 1).

Dialysis was significantly negatively associated with perceived severity (*β* = ‐ 0.21, *p* < 0.01) and positively associated with response efficacy (*β* = 0.16, *p* < 0.01) and intention (*β* = 0.10, *p* < 0.05). Self‐efficacy (*β* = 0.34, *p* < 0.001) and response efficacy (*β* = 0.27, *p* < 0.01) showed positive associations with intention; perceived vulnerability (*β* = 0.12, *p* < 0.05) and self‐efficacy (*β* = 0.22, *p* < 0.01), as well as intention (*β* = 0.26, *p* < 0.01), showed positive associations with CKD self‐management behavior. This study also explored alternative coding schemes about “dialysis stage,” and the results remain the same (see Supporting Figure S2).

## 6. Discussion

This study extends the application of PMT by utilizing SEM to elucidate the distinct cognitive mechanisms driving self‐management across CKD stages. Our findings reveal a critical stage‐dependent motivational shift: predialysis patients are primarily characterized by high threat appraisal (perceived severity), whereas dialysis patients are driven by high coping appraisal (response efficacy and self‐efficacy). This structural dichotomy suggests that the “engine” of self‐management evolves from fear‐based motivation to efficacy‐based control as the disease progresses.

### 6.1. Mechanisms of Threat Perception: Novelty vs. Normalization

Contrary to the intuitive assumption that advanced disease equates to higher perceived severity, our predialysis cohort reported significantly higher perceived severity than dialysis patients. This paradox can be explained by the psychological transition from uncertainty to normalization. For predialysis patients, the diagnosis represents a novel, looming threat characterized by “shock” and anxiety regarding the unknown progression to end‐stage renal disease [34]. The high perceived severity here reflects the acute stress of an anticipated catastrophe. In contrast, dialysis patients have survived the transition; the “threat” has materialized and become a routine part of daily life. This suggests a “response shift,” where patients recalibrate their internal standards to accommodate their condition, viewing dialysis not as an acute crisis but as a “new normal” [35]. Thus, lower perceived severity in the dialysis group reflects successful adaptation and illness integration rather than a lack of awareness.

### 6.2. Mechanisms of Coping Appraisal: The Role of Feedback Loops

A key finding of this study is that dialysis patients exhibited significantly higher response efficacy (belief that the behavior works) and self‐management intention than predialysis patients. We propose that this difference is structurally driven by disparities in healthcare exposure and feedback loops. Predialysis management (e.g., dietary restriction) often yields “invisible” results; patients may strictly adhere to a diet but feel no immediate physical difference, making it difficult to perceive the efficacy of their actions [36]. Conversely, dialysis patients operate in a high‐feedback environment. They interact with healthcare providers frequently (e.g., three times weekly) and receive immediate, objective data linking their behaviors to outcomes (e.g., interdialytic weight gain reflecting fluid control and monthly phosphate levels reflecting diet). This constant reinforcement validates the effectiveness of their self‐management, thereby boosting response efficacy. This explanation moves beyond symptom perception to highlight how clinical structural factors reinforce PMT constructs.

### 6.3. The Dominance of Efficacy in Driving Intention

Our SEM results confirm that across the continuum, coping appraisal (self‐efficacy and response efficacy) is a stronger predictor of intention than threat appraisal. This aligns with the revised PMT framework in chronic care, suggesting that while fear (threat) may initiate behavior, confidence (efficacy) sustains it [37, 38]. The strong correlation between self‐efficacy and intention underscores that “knowing what to do” is less critical than “believing one can do it.”

These stage‐specific mechanisms necessitate distinct intervention strategies. For predialysis patients, who suffer from high threat but low efficacy validation, interventions should focus on “tangibilizing” the benefits. Clinicians should use visual aids or biomarkers to demonstrate how current lifestyle changes are effectively delaying progression, thereby artificially creating the feedback loop that this stage lacks. For dialysis patients, who possess high response efficacy, care should focus on maintenance and reinforcement. Nurses should leverage the frequent clinical encounters to explicitly link clinical data (e.g., stable blood pressure) to the patient’s specific behaviors, reinforcing the existing belief that “what I do matters.” This differentiates care from generic education to targeted cognitive reinforcement based on the distinct PMT profiles of each stage.

## 7. Implications for Nursing Practice

Based directly on our SEM findings, which demonstrated that PMT cognitive appraisals significantly predict self‐management behaviors, nurses should pivot from generic behavioral education to cognition‐targeted assessments. Because our results identified constructs such as response efficacy and self‐efficacy as critical drivers of behavior, these specific cognitive pathways should form the basis of patient assessment. Recognizing the severe time constraints in routine clinical practice, administering full PMT questionnaires is not feasible. Instead, we recommend that renal nurses integrate rapid, 2‐to‐3‐question verbal screens (e.g., “Do you believe restricting fluids will actually help your kidneys?” to assess response efficacy) into routine clinical triage or electronic health records (EHR) to quickly identify patients’ motivational deficits.

Furthermore, derived directly from our study’s identification of distinct motivational pathways between disease stages, clinical counseling must be strictly stage‐specific. For predialysis patients, our data suggest that interventions must aggressively build early cognitive activation. Because these patients are tasked with delaying disease progression, nursing education should focus on translating abstract medical risks into tangible, immediate benefits of self‐management. Strategies such as nurse‐led motivational interviewing [39, 40] should be utilized specifically to target the exact cognitive gaps identified in our predialysis cohort.

Conversely, because our study specifically identified higher response efficacy among dialysis patients, nursing strategies for this group should shift toward leveraging and maintaining this existing confidence. Instead of basic psychoeducation, nurses should operationalize our findings by utilizing peer‐modeling and experiential learning [41], to reinforce the self‐efficacy required for complex, daily tasks like fluid and ion restriction. Ultimately, our findings strongly advocate for differentiating coaching protocols: predialysis coaching must focus on risk mitigation and cognitive activation, while dialysis‐stage coaching must focus on overcoming daily adherence barriers through continuous psychosocial reinforcement [40, 42].

### 7.1. Limitations

Several limitations warrant meaningful consideration. First, the cross‐sectional design precludes causal inference regarding the directional pathways between PMT constructs and self‐management behaviors. Longitudinal studies are needed to confirm how these motivational factors evolve over time. Second, participants were recruited via convenience sampling from a single urban district in Shanghai, with dialysis patients drawn from hospitals and predialysis patients from community centers. This sampling strategy, while reflecting the local tiered healthcare system, introduces potential selection bias regarding healthcare exposure and limits generalizability to rural or resource‐constrained settings. Third, unmeasured confounders such as illness duration, comorbidity burden, and dialysis vintage were not controlled. These factors are critical because long‐term patients often develop distinct coping mechanisms or “treatment fatigue” compared to newly diagnosed individuals, potentially influencing the observed PMT pathways. Furthermore, dialysis modality was not distinguished. Given that peritoneal dialysis and home hemodialysis require significantly higher self‐efficacy than in‐center hemodialysis, pooling these patients may have masked modality‐specific motivational drivers. Fourth, reliance on self‐report measures introduces susceptibility to recall and social desirability biases. Although we mitigated this by ensuring anonymous, private survey administration independent of the clinical team, self‐reported adherence is likely overestimated compared to objective clinical markers. Additionally, pragmatically excluding patients with sensory impairments (e.g., visual/hearing) limits the representativeness of this vulnerable subgroup. While data were collected in 2017–2018, the fundamental psychological constructs of threat and coping appraisal remain theoretically stable and relevant to current practice. Finally, we acknowledge the inherent limitation in comparing self‐management outcomes across groups due to the use of two distinct, stage‐specific instruments (CKD‐SM and SBSHP). While item mean scores were used to normalize the scales for analytical purposes, this adjustment does not resolve the underlying conceptual differences in construct coverage. Furthermore, because the two scales lack a sufficient set of identical anchor items, formal statistical tests for measurement invariance were not feasible. Consequently, our cross‐group comparisons should be interpreted as an evaluation of the structural relationships between PMT constructs and self‐management behaviors within their respective clinical contexts, rather than a direct comparison of absolute self‐management levels.

Collectively, these limitations underscore that while our findings robustly validate the structural applicability of PMT across CKD stages, cross‐group interpretations require caution. Specifically, the use of distinct, stage‐appropriate measurement tools without shared anchor items prevents the direct comparison of absolute self‐management scores. Thus, our conclusions focus on the comparative strength of motivational pathways rather than absolute behavioral levels. Future research should prioritize longitudinal designs and explore the development of unified measurement items to facilitate formal multigroup measurement invariance testing, thereby refining these stage‐specific motivational models.

## 8. Conclusions

This study elucidates the stage‐specific cognitive mechanisms driving self‐management in CKD through the lens of PMT. Our SEM demonstrates a critical motivational shift: While predialysis patients are primarily motivated by threat appraisal (perceived severity), dialysis patients are predominantly driven by coping appraisal (response efficacy and self‐efficacy). These distinct pathways underscore that nursing interventions must pivot from generic, fear‐based education to stage‐tailored, efficacy‐enhancing strategies. To concretely operationalize this, renal nurses should implement “efficacy‐focused feedback loops” during routine care. For instance, rather than merely instructing a dialysis patient on fluid restriction, nurses should explicitly and visually link the patient’s objective clinical data (e.g., reduced interdialytic weight gain or stabilized blood pressure) directly to their recent adherence efforts. This tangible reinforcement directly targets and elevates the response efficacy that our model identified as the strongest predictor of sustained self‐management behavior. Finally, while these findings robustly validate the structural applicability of PMT across the CKD continuum, they must be interpreted with methodological caution. Because distinct, stage‐specific instruments were utilized to capture the unique clinical realities of predialysis and dialysis patients, direct comparisons of absolute self‐management scores are inherently constrained. Therefore, these conclusions should be understood as reflecting differences in motivational pathways rather than absolute behavioral levels, and future longitudinal research is required to confirm the temporal stability of these cognitive shifts.

**Nomenclature**
CKD: Chronic kidney disease
PMT: Protection motivation theory
CFA: Confirmatory factor analysis
eGFR: Estimated glomerular filtration rate
SEM: Structural equation modeling
CKD‐SM: Chronic kidney disease self‐management
SBSHP: Self‐management behavior scale for hemodialysis patients
CNY: Chinese Yuan

## Author Contributions

Dayu Tang and Min Liang made substantial contributions to conception and design, or acquisition of data, or analysis, and interpretation of data and involved in drafting the manuscript or revising it critically for important intellectual content.

Dayu Tang, Min Liang, Wenyi Wang, and Yan Liang gave final approval of the version to be published. Each author should have participated sufficiently in the work to take public responsibility for appropriate portions of the content.

Wenyi Wang and Yan Liang agreed to be accountable for all aspects of the work in ensuring that questions related to the accuracy or integrity of any part of the work are appropriately investigated and resolved.

Dayu Tang and Min Liang should be indicated as co‐first authors.

## Funding

This study was funded by the National Natural Science Foundation of China, 72304071; Fudan University School of Nursing Research Fund, FNF202356.

## Disclosure

All the authors have verified that our submission adheres to the Journal’s statistical guidelines. We affirm that the methods employed in our data analyses are appropriately applied within the study design and context and that the statistical findings have been accurately implemented and interpreted. We accept responsibility for ensuring the appropriateness of the statistical approaches used in this submission.

## Conflicts of Interest

The authors declare no conflicts of interest.

## Supporting Information

Additional supporting information can be found online in the Supporting Information section.

## Supporting information


**Supporting Information** The supporting materials include the initial PMT scales for assessing CKD self‐management behaviors (Table S1), descriptive statistics of PMT factor items (Table S2), and a histogram indicating near‐normal distribution of self‐management behaviors (Figure S1). Fit indices for two CFA models are presented (Table S3), along with structural model results and alternative coding for “dialysis stage” (Figure S2).

## Data Availability

The data that support the findings of this study are available on request from the corresponding author. The data are not publicly available due to privacy or ethical restrictions.
