# Supplementary material for: Self‐Management Intentions and Behaviors Among CKD Patients at Predialysis and Dialysis Stages: A Cross‐Sectional Study Based on Protection Motivation Theory
Source: Nurs Res Pract. 2026 May 19;2026:6668409. doi: 10.1155/nrp/6668409 (PMC13189489; doi:10.1155/nrp/6668409)
Supplement: Supplementary file 1 — Supporting Information The supporting materials include the initial PMT scales for assessing CKD self‐management behaviors (Table S1), descriptive statistics of PMT factor items (Table S2), and a histogram indicating near‐normal distribution of self‐management behaviors (Figure S1). Fit indices for two CFA models are presented (Table S3), along with structural model results and alternative coding for “dialysis stage” (Figure S2). [file NRP-2026-6668409-s001.docx]

**Supplementary material**

**Table S1. The initial protection motivation theory (PMT) scales for self-management behaviors in CKD**

| **Item coding** | **Constructs & Item descriptions** | **Measurement** |
| --- | --- | --- |
| **Perceived severity**  Dear Participant, the following statements are related to your perception of the consequences of CKD. Please read the following statements carefully and choose the one that suits your opinion. | | For each item,  Strongly agree = 5  Agree = 4  No idea = 3  Disagree = 2  Strongly disagree = 1 |
| Seve1 | 1- Decreased kidney function is a serious problem for me. |  |
| Seve2 | 2- If I don't maintain treatment, my condition may relapse or worsen again even after it gets better. |  |
| Seve3 | 3. - Renal failure and complications will increase my family's burden and lead to poverty. |  |
| Seve4 | 4. - If my condition continues to deteriorate, it will be difficult for me to do anything. |  |
| **Perceived vulnerability**  Dear Participant, the following statements are related to your perception of the CKD risk. Please read the following statements carefully and determine to what extent you agree or disagree with them. | |  |
| Vuln1 | 1. - If I don't control my diet, my condition will worsen or I may even die. |  |
| Vuln2 | 2. - The risk of disease recurrence or complications is high for me without regulated self-management as directed by my doctor. |  |
| Vuln3 | 3. - The likelihood of my relapse is increased with insufficient exercise. |  |
| Vuln4 | 4. - The likelihood of my disease deterioration is increased with bad mood and anxiety. |  |
| **Self-Efficacy**  Dear Participant, the following statements are on your beliefs or whether you can perform CKD self-management confidently and successfully. Please read the following statements carefully and choose the option that suits your opinion. | |  |
| Self1 | 1- I can exercise moderately and effectively. |  |
| Self2 | 2- I can take medication as prescribed and self-monitor and perform self-care. |  |
| Self3 | 3. - I can remain optimistic about treatment of the disease. |  |
| Self4 | 4. - When the disease recurs or worsens, I can find ways to cope with it with the support of my doctor. |  |
| Self5 | 5. - I can prevent complications effectively. |  |
| Self6 | 6. I can take the initiative to communicate and interact with doctors to help me manage my kidney disease. |  |
| **Perceived response efficacy**  Dear Participant, the following statements relate to your view of activities that are effective in managing CKD. Please read the following statements carefully and choose the option that suits your opinion. | |  |
| Resf1 | 1- Knowledge of the disease can make me more adherent to treatment. |  |
| Resf2 | 2. - Following a low-protein diet as directed by my doctor can improve my quality of life. |  |
| Resf3 | 3. - Getting support from my family and friends can benefit treatment of the disease. |  |
| Resf4 | 4. - With regular follow-up and medication, my condition can be controlled to an ideal state. |  |
| **Perceived protection motivation (Intention)**  Think about the following questions and give the best answer. | |  |
| Inte1 | 1- I plan to take my medications as recommended by my doctor. |  |
| Inte2 | 2. - I plan to do a moderate amount of exercise (the amount recommended by my doctor) or rehabilitation activities each week. |  |
| Inte3 | 3. - I plan to gain more knowledge about the disease (through doctors, books, and the internet). |  |
| Inte4 | 4 - I plan to strictly control my diet and water intake in order to slow down the progression of my disease and improve my quality of life. |  |

We used maximum likelihood (ML) factor analysis with promax rotation to examine the dimensionality of the outcome expectations scales. Promax rotation was used when we expected the outcome expectations dimensions to correlate with one another^[[1]](#footnote-1)^. When ordinal variables have many categories (e.g., a 5- point (or more) Likert-type scales of agreement), and the variables show a moderate nonormality (i.e., skewness < 2 and kurtosis < 7), SEM analyses can be performed using the maximum likelihood (ML) approach^[[2]](#footnote-2)^. In this study, the PMT factors were measured using a 5- point Likert scales and the values for skewness and kurtosis were between -2 and +2, and between -7 and +7, respectively (see **Supplementary Table S2**). Besides, the values of self-management behaviors of CKD were continuous and was near normal distribution (see **Supplementary Figure S1**). Thus, the maximum likelihood (ML) approach was considered suitable for analysis in this study. Weighted least square mean and variance adjusted (WLSMV) estimator was also performed for EFA as an alternative approach to compare the results and model fit was assessed based on Xia & Yang’s recommendation^[[3]](#footnote-3)^.

**Table S2. Descriptive statistics of PMT factor items**

| **Items** | ***M*** | ***SD*** | **Skewness** | **Kurtosis** |
| --- | --- | --- | --- | --- |
| Seve1 | 4.10 | 1.117 | -1.295 | 3.893 |
| Seve2 | 4.29 | 0.839 | -1.466 | 5.887 |
| Seve3 | 4.01 | 1.089 | -1.087 | 3.436 |
| Seve4 | 4.26 | 0.825 | -1.153 | 4.406 |
| Vuln1 | 4.18 | 0.928 | -1.125 | 3.964 |
| Vuln2 | 4.18 | 0.926 | -1.148 | 4.046 |
| Vuln3 | 3.46 | 1.107 | -0.339 | 2.462 |
| Vuln4 | 3.73 | 1.083 | -0.506 | 2.553 |
| Self1 | 3.65 | 1.149 | -0.562 | 2.497 |
| Self2 | 4.35 | 0.774 | -1.144 | 4.334 |
| Self3 | 4.09 | 0.871 | -0.760 | 3.230 |
| Self4 | 3.91 | 0.928 | -0.617 | 2.832 |
| Self5 | 3.50 | 1.055 | -0.147 | 2.022 |
| Self6 | 3.99 | 0.998 | -0.704 | 2.600 |
| Resf1 | 4.48 | 0.666 | -1.064 | 3.552 |
| Resf2 | 4.22 | 0.849 | -1.051 | 3.946 |
| Resf3 | 4.20 | 0.848 | -1.135 | 4.452 |
| Resf4 | 4.39 | 0.700 | -1.086 | 4.607 |
| Inte1 | 4.58 | 0.661 | -1.753 | 6.829 |
| Inte2 | 4.21 | 0.930 | -1.118 | 3.805 |
| Inte3 | 4.01 | 1.007 | -0.985 | 3.711 |
| Inte4 | 4.26 | 0.794 | -1.026 | 4.211 |

**Figure S1. Histogram for self-management behavior of CKD**

For CFA, we examined two alternative models (Model A and Model B, see **Supplementary Table S3** ), Model A including all items (λ > 0.3) and Model B excluding items (λ > 0.3 on two or more factors). The results are presented in **Supplementary Table S3**. A post hoc modification was performed for both Model A and B according to model content.

**Table S3. Fit indices for alternative models from 5-factor CFA analyses**

| Model | ***χ²*** | *df* | TLI | CFI | AIC | BIC | SRMR | RMSEA (90% CI) |
| --- | --- | --- | --- | --- | --- | --- | --- | --- |
| Model A- before error correlation | 725.586* | 199 | 0.834 | 0.857 | 27630.078 | 27957.632 | 0.061 | 0.069 (0.064, 0.075) |
| Model B- before error correlation | 457.059* | 142 | 0.871 | 0.893 | 23662.484 | 23951.248 | 0.059 | 0.064 (0.057, 0.070) |
| Model A- after error correlation | 639.485* | 195 | 0.857 | 0.879 | 27526.344 | 27871.138 | 0.060 | 0.064 (0.059, 0.070) |
| **Model B- after error correlation** | **372.738*** | **138** | **0.901** | **0.920** | **23561.246** | **23867.251** | **0.058** | **0.056 (0.049, 0.062)** |

Notes:

1) Model A: f1 by seve1-seve4; f2 by vuln1-vuln4; f3 by resf1-resf4; f4 by self1-self6; f5 by inte1-inte4;

2) Model B: f1 by seve1-seve4; f2 by vuln3-vuln4; f3 by resf1-resf4; f4 by self2-self6; f5 by inte1-inte4;

3) Error correlation: inte2 with inte1; resf4 with resf3; inte1 with self2; seve4 with seve2;


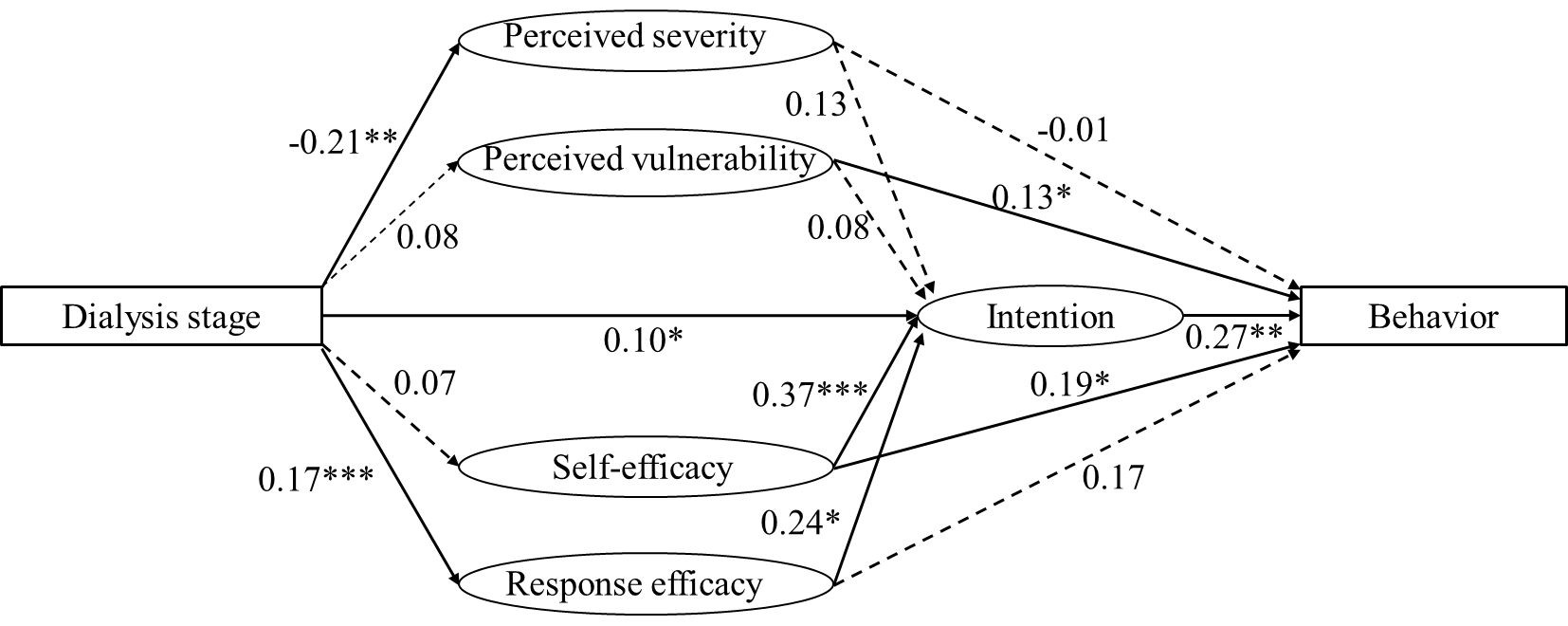


**Figure S2. Structural model results using an alternative approached to code “dialysis stage” (n = 526)**

*Notes: alternative coding scheme: “dialysis stage” (=1) as “stage 5 (complete renal failure) and receiving dialysis currently” (n = 216); “pre-dialysis stage” (=0) as “stage 1-4 and not receiving dialysis currently” ( n=310 ); excluding 24 who were in stage 5 (complete renal failure) but not receiving dialysis currently.*

The results of the structural model revealed a satisfactory fit to the data (χ2 = 567.413, df = 223, p < 0.001, χ2/ df = 2.544, RMSEA = 0.054 (90% CI 0.049, 0.060), CFI = 0.903, TLI = 0.874)

1. Noar SM, Myrick JG, Morales-Pico B, Thomas NE. Development and Validation of the Comprehensive Indoor Tanning Expectations Scale. Jama Dermatology 2014;150(5):512-21. [https://doi.org/](https://doi.org/10.1016/j.sc.2020.00372.)10.1001/jamadermatol.2013.9086. [↑](#footnote-ref-1)
2. Finney, S. J., & DiStefano, C. (2006). Nonnormal and Categorical Data in Structural Equation Modeling. In G. R. Hancock & R. O. Mueller (Eds.), Structural equation modeling: A second course (pp. 269-314). Greenwich, CT: IAP. [↑](#footnote-ref-2)
3. Xia Y, Yang Y. RMSEA, CFI, and TLI in structural equation modeling with ordered categorical data: The story they tell depends on the estimation methods. Behavior Research Methods 2019;51(1):409-28. [https://doi.org/](https://doi.org/10.1016/j.sc.2020.00372.)10.3758/s13428-018-1055-2. [↑](#footnote-ref-3)
